# Molecular mechanisms of circular RNA translation

**DOI:** 10.1038/s12276-024-01220-3

**Published:** 2024-06-14

**Authors:** Hyun Jung Hwang, Yoon Ki Kim

**Affiliations:** Department of Biological Sciences, Korea Advanced Institute of Science and Technology, Daejeon, 34141 Republic of Korea

**Keywords:** Ribosome, RNA decay

## Abstract

Circular RNAs (circRNAs) are covalently closed single-stranded RNAs without a 5′ cap structure and a 3′ poly(A) tail typically present in linear mRNAs of eukaryotic cells. CircRNAs are predominantly generated through a back-splicing process within the nucleus. CircRNAs have long been considered non-coding RNAs seemingly devoid of protein-coding potential. However, many recent studies have challenged this idea and have provided substantial evidence that a subset of circRNAs can associate with polysomes and indeed be translated. Therefore, in this review, we primarily highlight the 5’ cap-independent internal initiation of translation that occurs on circular RNAs. Several molecular features of circRNAs, including the internal ribosome entry site, *N*^6^-methyladenosine modification, and the exon junction complex deposited around the back-splicing junction after back-splicing event, play pivotal roles in their efficient internal translation. We also propose a possible relationship between the translatability of circRNAs and their stability, with a focus on nonsense-mediated mRNA decay and nonstop decay, both of which are well-characterized mRNA surveillance mechanisms. An in-depth understanding of circRNA translation will reshape and expand our current knowledge of proteomics.

## Circular RNAs: unlocking protein-coding potential beyond non-coding perception

This research delves into the intricate realm of circular RNAs (circRNAs), a kind of RNA molecule that forms a covalently closed circular structure, making it more stable than its linear counterparts. Despite being plentiful in cells, the role of circRNAs is largely a mystery. The researchers provide a summary of the various ways circRNAs are created and outline the different functions they can have. They also explore the molecular specifics of how circRNAs are translated and consider the potential interaction between this translation and their stability. The research is a review, summarizing and analyzing existing studies on the subject and highlighting the role and potential impact of circRNAs in the regulation of gene expression.

This summary was initially drafted using artificial intelligence, then revised and fact-checked by the author.

## Introduction

In eukaryotic cells, circular RNAs (circRNAs) are produced primarily by back-splicing events of pre-mRNAs^1^. Nascent pre-mRNAs are produced by RNA polymerase II and undergo multiple processing steps in the nucleus, including 5′-capping, 3′-polyadenylation, and splicing. Splicing removes introns and ligates consecutive neighboring exons to generate a mature messenger RNA (mRNA). Notably, not all splicing processes occur between exons located in close proximity in terms of nucleotide sequences. Specific features of nucleotides in introns flanking exons facilitate RNA looping, which causes the downstream 5′ splice site (splice donor) to be in close proximity to the upstream 3′ splice site (splice acceptor). These features include inverted repeats or nucleotide sequences that interact with RNA-binding proteins (RBPs) with dimerization ability. Spatial proximity between exon termini triggers an alternative noncanonical mode of splicing (termed back-splicing) and allows the biogenesis of circRNAs that are covalently closed structures and possess a head-to-tail connected single-stranded structure^2^. Due to the lack of both a 5′ cap and a 3′ poly(A) tail, circRNAs are generally resistant to exoribonuclease attack and exhibit greater stability than that of the cognate linear mRNAs with identical nucleotide sequences.

In this review, we provide an overview of the various methods for circRNA biogenesis and summarize the diverse molecular functions of circRNAs, including their roles as microRNA (miRNA) sponges, protein regulators, and templates for protein synthesis. Next, we examine the molecular details of circRNA translation and consider the possible relationship between circRNA translation and circRNA stability.

### CircRNA biogenesis

**Fig. 1:**
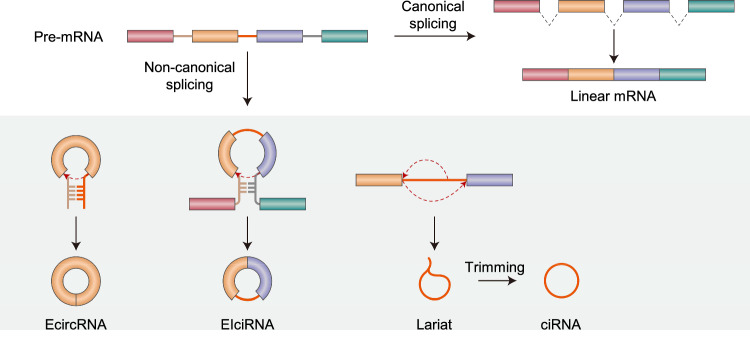
Biogenesis of circRNAs. CircRNA biogenesis from pre-mRNAs. EcircRNAs, EIciRNAs, and ciRNAs are generated via canonical splicing or back-splicing of pre-mRNAs.

Most eukaryotic circRNAs are produced via back-splicing^3–5^. Inverted Alu repeats or complementary sequences within two introns flanking the circularized exons cause the downstream 5′ splice site to come into close proximity to the upstream 3′ splice site and trigger intron pairing-driven back-splicing (Fig. 1)^4,6^. As a single gene may possess multiple introns, multiple circRNAs can be generated from a single gene as a consequence of alternative back-splicing using different 5′ and 3′ splice sites^4,7^. In the mouse heart, 1283 unique circRNAs were detected in six samples^8^. Almost half of the host genes were found to generate only one circRNA, while the other half of the host genes generated between two and nine circRNAs. The titin host gene can generate 38 distinct circRNAs via its I-band region, where complicated alternative splicing events take place^8^. Depending on the presence of exons and introns within the mature circRNA, circRNAs are classified into two groups (Fig. 1): exonic circRNAs (EcircRNAs), which are composed of one or more exons without introns; and exonic-intronic circRNAs (EIciRNAs), which contain one or more exons with at least one intron.

In addition to complementary sequences in the flanking introns, RBPs are another factor that can facilitate the biogenesis of both EcircRNAs and EIciRNAs. For example, muscleblind (MBL), a splicing factor, binds to both flanking introns and connects them in close proximity, thus promoting back-splicing^9^. As another example, quaking (QKI) binds to QKI-binding sites in flanking introns, and its dimerization brings the two flanking introns into close proximity, thus enhancing circRNA formation^10–12^. Like QKI, FUS and NOVA2 interact with the flanking introns of pre-mRNAs and play a role in circRNA biogenesis^13–16^. Conversely, certain RBPs contribute to the suppression of circRNA biogenesis by disrupting base pairing between complementary sequences in flanking introns. RBP-mediated inhibition of circRNA biogenesis is typified by A-to-I RNA editing, the posttranscriptional hydrolytic deamination of adenosine (A), in which an amino group at the C6 position in the double-stranded RNA (dsRNA) region is converted into inosine (I) by the enzymatic activity of adenosine deaminase acting on RNA (ADAR). Editing by ADAR destabilizes base pairing between inverted repeat sequences in the introns flanking the exons to be circularized and negatively regulates circRNA biogenesis^17,18^. Another RBP, DExH-box helicase 9 (DHX9), functions as an RNA helicase to unwind intron pairing between inverted repeat sequences. Accordingly, DHX9-depleted cells exhibit an increase in the number of expressed circRNAs^19^.

As an alternative means of circRNA biogenesis, lariat structures of introns excised from pre-mRNAs during nuclear splicing can be trimmed and matured into a subclass of circRNA known as circular intronic RNA (ciRNA; Fig. 1). The lariat introns that circularize tend to have C-rich sequences near the branch point, which presumably leads to inefficient debranching by the debranching enzyme^20^. In addition, when lariat structures are generated in exons as a consequence of alternative splicing, both EcircRNAs and EIciRNAs are generated. CircRNAs can also be generated from other RNA species in addition to pre-mRNAs. tRNA intronic circRNAs (tricRNAs) are generated during precursor tRNA (pre-tRNA) splicing^21–24^. In the process of pre-tRNA maturation, the tRNA-splicing endonuclease (TSEN) complex cleaves the bulge-helix-bulge motif of the pre-tRNA, and the resulting intron termini are ligated by the ligase RtcB to generate a tricRNA. In certain Archaea, circular intermediates harboring 16 S and 23 S rRNA are generated during ribosomal RNA processing^2^. Future studies should investigate the molecular roles of these circular intermediates.

Although diverse forms of circRNAs are produced as a consequence of circularization processes driven by intron pairing, RBPs, lariat structures, and TSEN-RtcB in eukaryotic cells, all types of circRNAs possess a common molecular feature—a covalently closed circular structure lacking both a 5′ cap and a 3′ poly(A) tail. Due to this property, circRNAs generally exhibit a longer half-life (ranging from 19 to 24 h) than do their cognate linear transcripts with identical nucleotide sequences (ranging from 4 to 7 h)^25^.

### Molecular functions of circRNAs

Thousands of distinct circRNAs are expressed in eukaryotic cells. The set of known molecular functions of these circRNAs is continually expanding, and these functions can be summarized as miRNA sponges, protein regulators, and templates for protein synthesis^1,20,26–35^.

First, certain circRNAs possess nucleotide sequence repeats that base pair with miRNAs, and these RNAs thus function as miRNA sponges^36–47^. Base pairing between circRNAs and miRNAs leads to the sequestration of functionally active miRNAs, and as a result, the mRNAs targeted by the miRNAs can escape miRNA-mediated gene silencing. For example, CDR1as (also known as ciRS-7) is highly expressed in both human and mouse brains and functions as a miRNA sponge^38^. CDR1as possesses 73 target sites for miR-7 binding^37^, and its expression causes midbrain defects that are similar to the phenotype observed when miR-7 is downregulated in zebrafish^43^. Certain circRNAs interact with multiple species of miRNAs. Circ-ITCH functions as a sponge that sequesters miR-7, miR-20a, and miR-214^39^, and circ-DAB1 sequesters miR-1270 and miR-944^36^. In addition, circCCDC66 functions as a miRNA sponge of miR-33b and miR-93, circ-Foxo3 binds to eight different miRNAs, and circHIPK3 harbors 18 potential binding sites for nine different miRNAs^45–47^. The identical circRNA may act as a sponge for distinct miRNAs in a tissue- or cell-type-specific manner. CircSLC8A1 acts as a miRNA sponge to sequester miR-130b and miR-494 in bladder cancer^42^ and miR-133a in cardiac hypertrophy^40^. In addition, circZNF609 acts as a miR-615-5p sponge in human retinal endothelial cells^41^ and as a miR-150-5p sponge in Hirschsprung’s disease^44^.

CircRNAs can also serve as protein decoys, adaptors, scaffolds, or sponges to influence protein function^1,20,26–35^. These molecular roles include (i) transcriptional or splicing regulation through R-loop formation by circRNAs, (ii) stem cell maintenance or PKR inhibition through short stem-loop structures in circRNAs, (iii) gene regulation by sequestering or outcompeting functional proteins, (iv) regulation of biological activities through the formation of complexes containing the circRNA and proteins, and (v) protein synthesis using the circRNA as a template for translation. In addition to the abovementioned functions, future research is expected to reveal an even wider array of molecular functions for circRNAs.

## Circular RNA translation

**Fig. 2:**
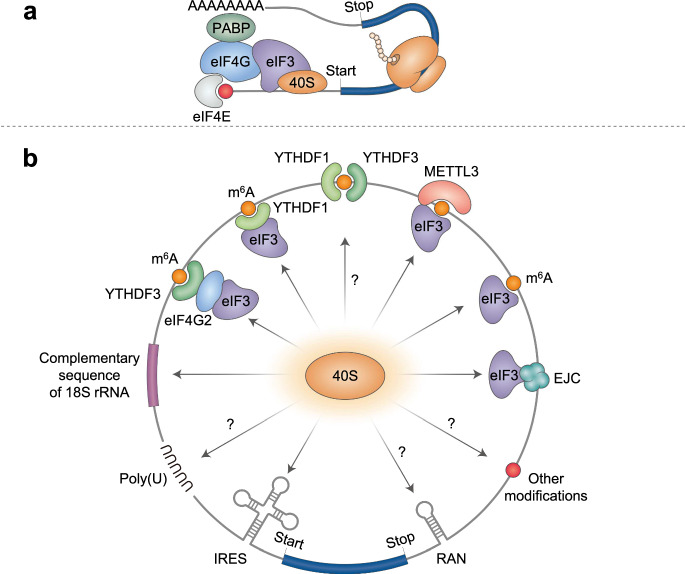
Molecular mechanisms for canonical cap-dependent translation initiation and internal initiation of translation. **a** Canonical cap-dependent translation initiation. The molecular axis of eIF4E-eIF4G-eIF3-40S ribosome is critical for 5′-cap-dependent translation initiation. The interaction between eIF4G and PABP1 results in the formation of a looped structure, which is favorable for translation initiation. **b** Various modes of internal translation initiation of circRNAs. Various molecular features of circRNAs contribute to internal entry of the ribosome to initiate translation. These features include (i) canonical IRESs; (ii) IRES-like sequences or elements such as m^6^A, poly(U) sequence motifs, complementary sequences to 18 S rRNA, and tandem repeats of short nucleotides that can induce RAN translation; and (iii) the EJC deposited on the circRNA after back-splicing. The features for which the molecular mechanism underlying their involvement in ribosome recruitment is not clear are marked with a question mark.

Most eukaryotic mRNAs possess a 5′-cap and a 3′-poly(A) tail, both of which play critical roles in ribosome recruitment to the mRNA. According to the widely accepted model for canonical cap-dependent translation (Fig. 2a), the 5′-cap is recognized by eukaryotic translation initiation factor (eIF) 4E, the major cytoplasmic cap-binding protein^48,49^. Then, eIF4E at the 5′-cap of mRNA sequentially recruits eIF4G (a molecular scaffold with multiple docking sites for other eIFs) and the eIF3 complex. Concomitantly, the 3′-poly(A) tail associates with the cytoplasmic poly(A)-binding protein (PABP1). PABP1 makes direct contact with eIF4G and helps bring the 5′- and 3′-ends into close proximity, forming a translationally favorable loop structure. Eventually, the small subunit of the ribosome (40 S) is loaded onto the 5′-end of the mRNA and initiates the scanning process to search for the translation initiation codon (AUG) in the proper context. When the 40 S ribosome locates the AUG codon, the large subunit of the ribosome (60 S) joins the 40 S ribosome complex, and protein synthesis begins. In addition to the canonical cap-dependent translation mentioned above, ribosomes can be recruited to mRNAs in various ways. In principle, as long as the 40 S ribosome is loaded onto an mRNA, it possesses the potential to initiate protein synthesis following 40 S scanning and 60 S joining.

For circRNAs, internal loading of the ribosome is the only means to initiate translation, as circRNAs lack a 5′-cap and a 3′-poly(A) tail. Many recent studies have provided compelling evidence supporting the internal initiation of translation on circRNAs^50–61^. We explore various molecular mechanisms that drive the internal entry of ribosomes to enable protein synthesis from circRNAs (Fig. 2b).

### Internal initiation of translation mediated by internal ribosome entry sites (IRESs)

Canonical IRESs were initially discovered in the 5′-untranslated region (5′UTR) of genomic RNA of picornavirus^62,63^. These are typically defined as RNA secondary or tertiary structures that promote mRNA translation by recruiting the 40 S ribosome to the 5′UTR in a cap-independent manner^64,65^. Subsequent studies revealed that efficient IRES-dependent translation requires a subset of general eIFs and IRES-specific trans-acting factors. Notably, many endogenous eukaryotic mRNAs use IRESs for protein synthesis, even under conditions in which cap-dependent translation is compromised. Experimentally validated cellular and viral IRESs in eukaryotic cells are available in IRESbase (http://reprod.njmu.edu.cn/cgi-bin/iresbase/index.php)^66^. A caveat in the IRES field is that many RNA sequences or elements without distinctive RNA secondary or tertiary structures, such as *N*^6^-methyladenosine (m^6^A) RNA modifications, drive the internal entry of ribosomes, thus enabling internal initiation of translation. Therefore, regardless of the presence of RNA structures, canonical IRESs and any other IRES-like sequences or elements that can promote the internal entry of ribosomes are hereafter referred to as IRESs.

Various cellular, viral, and artificial IRESs have been identified using high-throughput screening systems. Chen et al. identified approximately 17,000 sequences with putative IRES activity using a high-throughput screening assay with a split-eGFP circRNA reporter^67^. In-depth analysis of RNA structures and sequences revealed that a complementary sequence for 18 S rRNA and a stem‒loop-structured RNA element (SuRE) located approximately 40–60 nucleotides downstream of the first nucleotide of the IRES promote circRNA translation. Weingarten-Gabbay et al. performed high-throughput screening using a dicistronic reporter system to search for IRESs^68^. Although they did not directly apply their findings to circRNA translation, they observed that a distinct secondary structure, a poly(U) sequence motif, and a complementary sequence for 18 S rRNA in the intercistronic region serve as functional IRESs. In addition, for the purpose of identifying short IRES-like elements, Fan et al. developed a GFP-expressing circRNA reporter library harboring artificially designed random 10-mer sequences in the UTR and conducted high-throughput screening^53^. Through subsequent bioinformatic analysis, these researchers identified 97 hexamers that function as IRES-like elements. These hexamers accounted for approximately 2% of the entire pool of hexamers (4^6^ = 4096). Based on the lengths of the endogenous circRNAs and statistical calculations, Fan et al. proposed that most circRNAs may coincidentally contain IRES-like elements, suggesting a potentially greater occurrence of circRNA translation than previously appreciated.

Independent researchers have also identified IRESs in circRNAs^57,60,67,69–79^. Legnini et al. reported that the UTR of circZNF609 serves as an IRES^54^. Notably, this group demonstrated that the IRES in circZNF609 functions in a splicing-dependent manner, a unique feature when compared to canonical IRESs. Pamudurti et al. revealed that circMbl is translated in a cap-independent manner. It should also be noted that both the UTR of circMbl and its reverse sequence promote circRNA translation, suggesting that a distinct RNA structure rather than the RNA sequence itself may drive the internal initiation of circMbl. Zhang et al. identified circPINTexon2, a circRNA generated from exon 2 of a long intergenic non-coding RNA^60^. CircPINTexon2 contains a functionally active IRES upstream of a short open reading frame (ORF) encoding 87 amino acids (aa).

Recently, it was proposed that the pathogenic expansion of tandem repeats of short nucleotides, which is associated with many human genetic diseases^80^, can drive internal initiation of translation in certain cases^81–83^. In general, expanded repeat RNAs can drive noncanonical translation, referred to as repeat-associated non-AUG (RAN) translation, in which translation initiation occurs at the near-cognate AUG codon rather than at the canonical AUG codon^83^. RAN translation of CGG repeats is initiated within the repeat and in the absence of any near-cognate codons^83^. In addition, RAN translation on GGGGCC repeats of C9ORF72 RNA can be initiated in a cap-independent manner depending on the location and context of the expanded repeats^82^. Indeed, Wang et al. revealed that the GGGGCC repeat-containing intron of the C9ORF72 gene is processed and matured into a ciRNA and undergoes RAN translation to produce toxic dipeptide repeat-containing polypeptides^81^. Further molecular details of RAN translation of circRNAs and the biological impacts of this process should be addressed in future studies.

### Internal initiation of translation mediated by m^6^A modification

m^6^A is the most prevalent internal modification in mammalian mRNAs. This modification affects almost all aspects of mRNA metabolism, including transcription, pre-mRNA splicing, nuclear export, mRNA stability, and translation^84–89^. Consequently, diverse cellular and physiological processes are affected by m^6^A modifications. Tens of m^6^A-recognizing RBPs, including YTHDF1–3, eIF3, and METTL3, have been characterized.

m^6^A contributes to the efficient translation of linear mRNAs in a number of ways. When the 5′UTR of an mRNA contains m^6^A, eIF3 directly recognizes the m^6^A site and thereby promotes translation initiation. Notably, this mode of translation occurs in a 5′-cap-independent but 5′-end-dependent manner^90^. However, considering that the hepatitis C viral IRES provides a platform for the recruitment of eIF3 and drives the internal entry of ribosomes^64,65^, it is possible that a subset of circRNAs containing m^6^A may employ this strategy to recruit ribosomes.

The m^6^A located in the 3′UTR of a linear mRNA can also promote translation in several different ways. YTHDF1 directly recognizes m^6^A sites in the 3′UTR and recruits eIF3 to promote translation, possibly further stabilizing a translationally favorable loop structure^91^. YTHDF3 is also known to recognize m^6^A sites in 3′UTRs, thus promoting the translation of linear mRNAs in either a YTHDF1-dependent or YTHDF1-independent manner^92,93^. Furthermore, METTL3, an m^6^A writer protein, contributes to increased translation of linear mRNAs via its physical interaction with eIF3h, a component of the eIF3 complex^94,95^. Artificial tethering of a catalytically inactive form of METTL3 to the 3′UTR of a linear reporter mRNA is sufficient for the increase in translation, indicating that the observed increase is not dependent on the methyltransferase activity of METTL3. Electron microscopy revealed that the METTL3-eIF3 interaction facilitates the formation of a translationally favorable loop structure in mRNA. Given that eIF3 binding to mRNA is sufficient for the internal entry of ribosomes, as demonstrated for the hepatitis C viral IRES^64,65^, YTHDF1-, YTHDF3-, or METTL3-mediated internal initiation of translation may occur on m^6^A-containing circRNAs. Indeed, YTHDF3 recognizes m^6^A within circRNAs and recruits eIF4G2 and eIF3, ultimately leading to the internal entry of 40 S ribosomes^59^. It should also be noted that in addition to m^6^A, other types of mRNA modifications, such as A-to-I RNA editing, may contribute to internal ribosomal entry^96^, which is an intriguing topic for future research.

### Internal initiation of translation mediated by eIF4A3 or the exon junction complex

Internal loading of 40 S ribosomes onto circRNAs can be mediated by the exon junction complex (EJC)^61^, a specialized multiprotein complex deposited approximately 20–24 nucleotides upstream of each exon‒exon junction after a canonical nuclear splicing event^97–99^. EJC loading is initiated by the formation of a stable trimeric complex via the locking of eIF4A3 onto the mRNA with the help of Y14 (also known as RNA-binding motif 8 A) and mago-nashi homolog (MAGOH). Subsequently, metastatic lymph node 51 (MLN51, also known as barentsz or CASC3) joins the complex to form a stable tetrameric core. Once loaded onto a mature mRNA, the EJC plays multifaceted roles in various gene regulatory mechanisms, including pre-mRNA splicing, mRNA export, translation, and mRNA stability.

Several studies have implicated the possible role of the EJC in the internal translation of circRNAs. For example, when a putative IRES present in a circRNA was artificially inserted into the intercistronic region of a dicistronic reporter mRNA, it drove the translation of the second cistron of the dicistronic reporter mRNA^54^. Notably, the insertion of the putative IRES with an endogenous intronic sequence into the intercistronic region further facilitated the translation of the second cistron^54^, suggesting that the EJC loaded onto the intercistronic region after splicing of the endogenous intronic sequence may act cooperatively with the IRES and contribute to the internal initiation of translation. In another study, a complementary sequence to 18 S rRNA and a SuRE located approximately 40–60 nucleotides downstream of the first nucleotide of the IRES present within a circRNA was demonstrated to facilitate translation of the circRNA^67^. Notably, the IRESs were located predominantly near the back-splicing junction (BSJ), suggesting a possible interplay between the IRES and the EJC loaded in close proximity to the BSJ^67^.

Recently, a direct role for the EJC in circRNA translation has been reported^61^. As back-splicing to generate circRNAs is an alternative form of canonical nuclear splicing, it is plausible that the EJC is loaded onto the BSJ on circRNAs. It has been demonstrated that the EJC deposited on a circRNA can recruit the eIF3 complex via an interaction between eIF4A3 and eIF3g (a subunit of the eIF3 complex)^61^. This finding suggests that the EJC functions as a molecular scaffold to recruit the eIF3 complex and 40 S ribosome to circRNAs, thereby driving the internal initiation of translation. In support of this idea, polysome fractionation followed by transcriptomic analysis revealed that the polysomal association of endogenous circRNAs depends upon eIF4A3^61^. It should also be noted that eIF4A3 alone possesses the intrinsic capability to initiate internal translation in an eIF3-dependent manner, and this capacity is further augmented by the presence of other EJC components. Therefore, these observations suggest that both the EJC loaded onto the circRNA after back-splicing and free eIF4A3 associated with the circRNA possess the ability to initiate internal circRNA translation. Notably, Lin et al. ^100^ reported that a subset of endogenous circRNAs is associated with actively translating ribosomes and that the internal translation of circSDHAF2 is mediated by the EJC deposited onto the circRNA after back-splicing.

The internal initiation of circRNA translation through the interaction between the EJC and eIF3 may be modulated by several factors. Song et al. assessed the effects of 43 Drosophila eIFs on the internal translation of Drosophila circSfl^101^. They observed that the eIF3 complex increases the translation efficiency of circSfl. In contrast, eIF3j, a component of the eIF3 complex, triggered the dissociation of the eIF3 complex from the circRNA, thereby functioning as an inhibitory factor of circRNA translation. Considering that the C-terminal region of eIF3j is critical for its inhibitory function, it would be interesting to determine whether eIF3j affects the interaction between the EJC and the eIF3 complex. Alternatively, EJC deposition on circRNAs during back-splicing may be affected. For instance, eIF4A3 is phosphorylated by cyclin-dependent protein kinases 1 and 2 in a cell cycle-dependent manner^102^. As the phosphorylation site is located within the RNA-binding motif, phosphorylation can cause dissociation of the EJC from mRNAs or inefficient loading of the EJC onto mRNAs. Thus, the internal translation of circRNAs mediated by the interaction between the EJC and the eIF3 complex could be affected by the phosphorylation status of eIF4A3 in a cell cycle-dependent manner.

## Diverse protein isoforms translated from circular RNAs

**Fig. 3:**
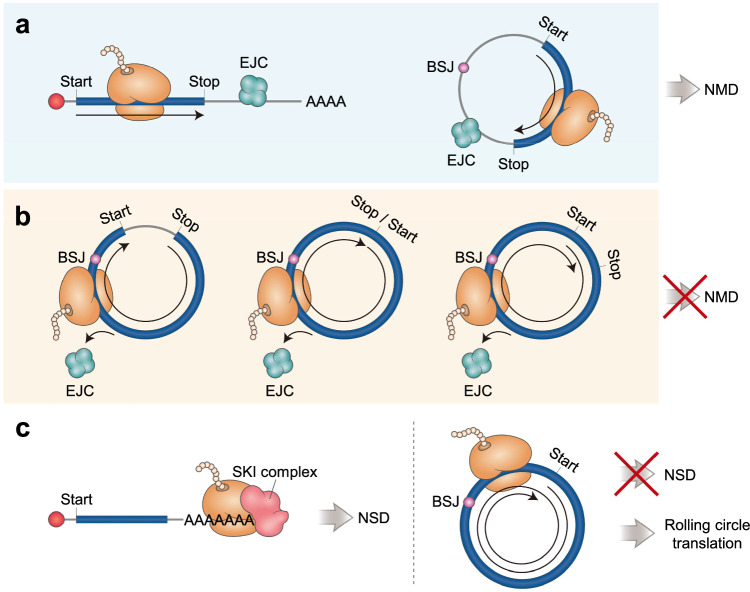
Various types of ORFs in circRNAs and their roles in circRNA stability. **a** NMD of linear mRNA and circRNA. The presence of the EJCs sufficiently downstream of the stop codon induces rapid degradation of linear mRNAs via the NMD pathway (left). Similarly, when a circRNA contains an EJC (at the BSJ) sufficiently downstream of the stop codon, it would be targeted for NMD (left). **b** Diverse ORFs in circRNAs depending on the relative positions of translation start codons, stop codons, and the BSJ. When the EJC is deposited onto the ORF in a circRNA, it would be displaced from the circRNA by elongating ribosomes. Consequently, the circRNA would evade NMD and be stabilized. **c** Rolling circle translation of circRNAs lacking stop codons. Whereas a linear mRNA lacking stop codons is rapidly degraded via the NSD pathway (left), a circRNA lacking stop codons would be subject to rolling circle translation (right).

After the ribosome is loaded onto a circRNA, it may initiate protein synthesis, provided that the circRNA has an ORF. As circRNAs are generated by back-splicing, the proteins translated from circRNAs could have identical, similar, or chimeric forms in terms of their amino acid sequences compared to those of the proteins synthesized from precursor mRNAs of circRNAs. Therefore, diverse protein isoforms can be generated via internal translation of circRNAs depending on the relative positions of the translation start codons (AUG or non-AUG codons), translation stop codons (UAA, UAG, and UGA), and BSJ (Fig. 3). It should also be noted that these relative positions can affect circRNA stability.

### Translation start and stop on circRNAs

CircRNAs can generate multiple proteins when they have more than one start codon or when they use a non-AUG start codon. The translatable circZNF609 can produce at least three proteins that can be detected using western blotting^54,103^. This circRNA has at least two ORFs generated by circularization via back-splicing. The stop codon is located upstream of the start codon and three nucleotides downstream of the BSJ. Therefore, new chimeric proteins can be synthesized from these ORFs. In addition to circZNF609, diverse circRNAs are known to produce new chimeric proteins due to the new ORFs generated by back-splicing^71,73–75,78^. For instance, back-splicing of exons 3–6 of the SMO gene produces circSMO, which contains a new ORF encoding a chimeric protein of 193 amino acids^75^.

As an alternative method for generating chimeric proteins, an in-frame stop codon can be positioned after the second round of translation. Ribosome profiling revealed 1879 translatable putative circRNAs in glioblastoma and normal brain tissue samples. Among these, circular E-cadherin RNA (circ-E-Cad) was found to have no in-frame stop codon^77^. However, circ-E-Cad generated a 254 aa protein from 733 nucleotides. In this protein, 240 aa were synthesized during the first-round translation of the ORF, and the remaining 14 aa were synthesized during the second-round translation of the ORF. Therefore, circ-E-Cad contains a unique 14 amino acid tail generated through a natural frameshift during the second round of translation, as an in-frame stop codon appears only after the second round of translation.

As observed in many linear mRNAs^83^, translation initiation at non-AUG start codons has been reported in circRNAs. For instance, CircE7, which was identified in papillomaviruses, can be translated despite the lack of an ATG start codon^104^. Circ-PAPOLA, lacking an annotated AUG start codon, is also translated^105^. Considering the non-AUG translation initiation observed for circRNAs, it is plausible that other phenomena that occur during the translation of linear mRNAs, such as ribosome read-through and reinitiation, could also occur during circRNA translation. In particular, given that the EJC can be directly associated with mRNA stability, the relative positions of the BSJ and stop codons in a circRNA may affect the stability of the circRNA. In the case of linear mRNAs, most EJCs loaded onto mRNAs after splicing are displaced from the mRNAs by elongating ribosomes. If the EJC remains on an mRNA even after translation termination (if the EJC is located sufficiently downstream of the stop codon), the mRNA is recognized as a faulty transcript and is rapidly degraded via the mRNA surveillance pathway, nonsense-mediated mRNA decay (NMD^106–109^; Fig. 3a, left). Similarly, in the case of circRNAs, if the BSJ is located sufficiently downstream of the stop codon, the EJC remains near the BSJ even after translation termination, and the circRNA would be recognized as a faulty transcript and rapidly eliminated via NMD (Fig. 3a, right). Otherwise, if the BSJ is located upstream of the stop codon, the EJC near the BSJ will be displaced by elongating ribosomes, and the circRNA would escape NMD and be stabilized (Fig. 3b). Thus, the relative positions of the BSJ and stop codon in the ORF within circRNAs may be a critical factor determining circRNA stability.

In some cases, the AUG start codon can overlap with the stop codons in a circRNA. For instance, circ-AKT3 has a UAAUGA sequence (the start codon is underlined) and produces a protein of 174 amino acids^72^. In this circRNA, the AUG start codon overlaps with the stop codon. Similarly, circ-SHPRH contains a UGAUGA sequence (the start codon is underlined) and produces a 17 kDa protein^70^. Furthermore, the circRNA generated from rice yellow mottle sobemovirus has a short translatable ORF (220 nucleotides long) with overlapping start and stop codons (UGAUGA; the start codon is underlined). Intriguingly, this circRNA synthesizes proteins of 16 to 39 kDa, corresponding to up to five rounds of translation through its UGAUGA sequence, thus providing evidence for the occurrence of translation read-through on circRNAs^110^.

### Translation start and nonstop on circRNAs

In general, when a linear mRNA lacks stop codons, the ribosome elongates to the end of the mRNA and exhibits a unique structure with an empty ribosomal A site. In this situation, the mRNA expresses a C-terminally extended protein that can be potentially toxic to normal cellular functions. To minimize these potential toxic effects, eukaryotic cells have evolved nonstop decay (NSD)^108,109,111–113^, an mRNA surveillance mechanism by which transcripts lacking stop codons are recognized as faulty transcripts and are rapidly degraded (Fig. 3c, left).

What happens when circRNAs lack an in-frame stop codon? In this scenario, internally loaded ribosomes would initiate translation and continue to synthesize proteins endlessly from infinite ORFs, a process known as rolling circle translation (Fig. 3c, right). Rolling circle translation results in the synthesis of high-molecular-weight proteins of multimeric forms corresponding to repeats of identical ORF sequences. As a consequence of rolling circle translation, circRNAs lacking stop codons would evade NSD, which triggers rapid degradation of linear mRNAs lacking stop codons.

Artificially generated circRNAs can undergo rolling circle translation^114,115^. For instance, the artificially designed circRtn4 produces multimeric polypeptides from an infinite ORF^116^. In addition to artificially generated circRNAs, a subset of endogenous circRNAs, including circALTO1, circ-E-Cad, circEGFR, circCORO1C, and circASPH, have an infinite ORF without stop codons and are capable of rolling circle translation^77,117–119^. For example, in Merkel cell polyomavirus, circALTO1, which contains 762 nucleotides harboring a start codon but lacking a stop codon, is capable of rolling circle translation^119^. A recent study also underscored the potential importance of A-to-I RNA editing in the context of rolling circle translation^96^. Two circRNAs generated through back-splicing of exon 12 to either exon 7 (12 → 7 circRNA) or exon 10 (12 → 10 circRNA) of the human tau gene can be translated via the rolling circle mechanism^96^. These two circRNAs lack stop codons. The 12 → 7 circRNA contains one start codon and expresses a multimeric protein via rolling circle translation. The 12 → 10 circRNA is targeted for A-to-I RNA editing mediated by ADAR. As a result, a new start codon is generated by the editing of AUA (Ile codon) to AUI (a cognate start codon), allowing the 12 → 10 circRNA to be translated via the rolling circle mechanism. The molecular details underlying the internal initiation of translation at the AUI codon should be explored in future studies. It should also be noted that the proteins synthesis via rolling circle translation are not infinite. Liu et al. suggested the existence of a programmed -1 ribosomal frameshift (-1PRF) mechanism during rolling circle translation. To terminate the otherwise endless translation, an out-of-frame stop codon is generated via the -1PRF mechanism^120,121^, suggesting that rolling circle translation could be terminated in the end.

## Conclusions

Accumulating evidence supports the translatability of endogenous circRNAs through internal loading of ribosomes. Furthermore, the roles of proteins and peptides translated from circRNAs are expanding dramatically, ushering in a new era of research on a variety of biological and physiological events, including cancer, stemness, and pluripotency^122^. However, many unanswered questions regarding circRNA translation remain to be addressed. First, as the internal translation of circRNAs is generally inefficient compared to the canonical cap-dependent translation of linear mRNAs, it is challenging to detect protein and peptide products generated from circRNAs due to technical limitations. Therefore, there is an unmet need to develop methodologies more sensitive than the current proteomic techniques to discover unique proteins and peptides translated from circRNAs. Second, many unique proteins and peptides can be generated from circRNAs. It is crucial to determine whether the proteins and peptides generated from circRNAs play important roles in biological and physiological processes or if they are simply erroneous proteins or byproducts that are rapidly degraded via the protein surveillance pathway. Finally, the detailed molecular mechanisms underlying internal ribosome recruitment to circRNAs should be elucidated in more intensive studies. For instance, when identical IRESs or IRES-like elements are present in both linear mRNAs and circRNAs, the ability of these mRNAs to recruit ribosomes would differ because circRNAs can form their own internal secondary or tertiary structures, unlike linear mRNAs with the identical nucleotide sequences^123^. Gaining a comprehensive understanding of circRNA structures using computational and bioinformatics approaches will answer these questions in the future.
